# Two Novel *NF1* Pathogenic Variants Causing the Creation of a New Splice Site in Patients With Neurofibromatosis Type I

**DOI:** 10.3389/fgene.2019.00762

**Published:** 2019-08-22

**Authors:** Vita Setrajcic Dragos, Ana Blatnik, Gasper Klancar, Vida Stegel, Mateja Krajc, Olga Blatnik, Srdjan Novakovic

**Affiliations:** ^1^Department of Molecular Diagnostics, Institute of Oncology Ljubljana, Ljubljana, Slovenia; ^2^Cancer Genetics Clinic, Institute of Oncology Ljubljana, Ljubljana, Slovenia; ^3^Department of Pathology, Institute of Oncology Ljubljana, Ljubljana, Slovenia

**Keywords:** NF1, splicing, mRNA, functional analysis, NGS, neurofibromatosis type I, splicing alteration, variant of uncertain significance

## Abstract

Neurofibromatosis type I (NF1) is one of the most common autosomal dominant disorders, since the estimated incidence is one in 3,500 births. In this study, we present bioinformatical and functional characterization of two novel splicing *NF1* variants, detected in NF1 patients. Patient 1, carrying *NF1*:c.122A>T, which introduces a new exonic 5’ donor splice site, was diagnosed with hormone-positive, Her-2-negative breast cancer at the age of 47. She had an atypical presentation of NF1, with few café-au-lait spots and no Lisch nodules. Patient developed a hemothorax due to subclavian artery rupture, which has previously been described as an extremely rare complication of NF1. Patient 2, carrying *NF1*:c.7395-17T>G that creates a new intronic 3’ acceptor splice site, had quite a typical clinical presentation of NF1: formations on her tongue in the region of her left metacarpal bones and on her left foot, plexiform neurofibroma in her pelvis, several café-au-lait spots, and axillary freckling. She was also diagnosed with cognitive impairment. In the report, we are presenting two novel variants which were successfully classified based on NGS and mRNA analysis. Based on results of mRNA analysis, both variants were classified as likely pathogenic according to ACMG guidelines applying evidence categories PS3, PM2, PP3, and PP1 supporting. By characterizing those two novel *NF1* splicing variants, we have confirmed the neurofibromatosis type I phenotype in the two probands.

## Introduction

Neurofibromatosis type 1 (NF1) (OMIM# 162200) is a disease caused by loss of function mutations in the *NF1* gene. NF1 is inherited in an autosomal dominant manner with complete penetrance in adulthood. Patients with neurofibromatosis type 1 are at higher risk of developing malignancies, such as breast cancer, gastrointestinal stromal tumors, and malignant peripheral nerve sheath tumors (Friedman, 1993). A study based in Finland showed an estimated lifetime cancer risk of 59.6% in patients who harbor *NF1* gene mutation (Uusitalo et al., 2016). Recent advances in sequencing and screening of NF1 gene have increased the number of detected variants, leading to the increased yield of NF1 diagnosis. As of September 2018, 3,786 *NF1* variants were submitted to ClinVar (Landrum et al., 2018), out of which 1,594 (43%) were classified as variants of uncertain clinical significance (VUS). Therefore, a big proportion of *NF1* variants, detected in patients’ DNA, cannot be clearly classified as either pathogenic or benign. Precise classification of variants is the key to a proper clinical management; thus, VUS are highly problematic since they can cause confusion among patients and professionals (Hoffman-Andrews, 2017). Classification of novel frameshift, nonsense, and consensus splice site variants is quite straightforward, as they lead to a complete loss of gene product by nonsense mediated decay and therefore loss of functional NF1 protein. Particularly challenging to classify are missense, silent, and intronic variants, since they can either affect protein folding, impair the important functional domain, or can interrupt normal mRNA splicing. Variants that affect consensus splice sites can cause complete exon skipping, intron retention, activation of cryptic splice site, or introduction of a new splice site (Scotti and Swanson, 2016). Since up to 30% of *NF1* pathogenic variants affect splicing with a high frequency of splice mutations outside of the AG/GT 5’ and 3’ splice sites, some laboratories analyze the gene using cDNA based Sanger sequencing (Messiaen et al., 2000). Alternatively, it is possible to perform gDNA sequencing first. If a variant is detected which is suspected to affect splicing, it is crucial to perform additional tests such as functional mRNA analysis in order to determine the variant’s impact on splicing. Here, we report two novel variants that have been classified based on functional mRNA analysis.

## Case Presentation

### Patient 1 (Harboring *NF1*:c.122A > T)

The first patient was referred to our cancer genetics clinic at the age of 54 by her oncologist with the clinical diagnosis of neurofibromatosis type I. The patient developed a hormone-positive, Her-2-negative breast cancer aged 47. At diagnosis, she underwent a mastectomy, followed by chemotherapy (fluorouracil, epirubicin, cyclophosphamide) and adjuvant hormonal therapy with tamoxifen. At age 48, she was diagnosed with an ovarian tumor, which proved to be a mucinous cystadenoma, and a bilateral adnexectomy was performed. Subsequently, tamoxifen was replaced with letrozole and later with anastrozole. The patient has no signs of breast cancer relapse. Aged 55, she developed a massive hemothorax due to right subclavian artery rupture and hemorrhage. A thoracotomy was performed. After the procedure, the patient developed pulmonary embolism but recovered with no adverse sequelae. She has recently been diagnosed with osteoporosis.

**Figure 1:**
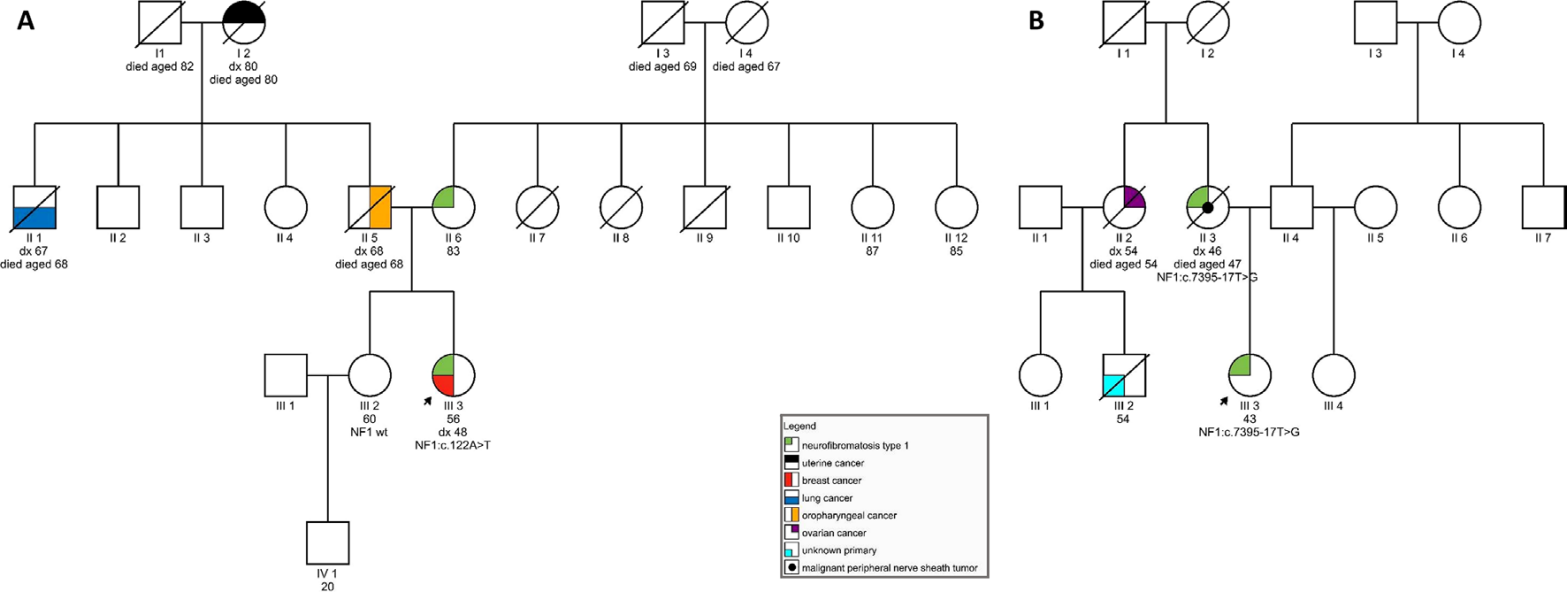
**(A)** Pedigree of a family carrying variant *NF1:c.122A > T*; **(B)** Pedigree of a family harboring *NF1:c.7395-17T > G*.

On examination at our clinic, she had less than six café-au-lait spots. In addition, axillary freckling was observed. She had numerous cutaneous fibromas, which gave the appearance of neurofibromas—four had been excised in the past and were diagnosed as fibroepithelial polyps. She also had subcutaneous formations, some of which had been excised in the past but were shown to be lipomas. Her ophthalmological examination was normal, and no Lisch nodules were observed. She had no musculoskeletal findings, typical of neurofibromatosis. In the patient’s family, her mother was said to have skin manifestations, similar to those seen in the daughter. The patient’s sister was also seen at our clinic and had no signs of NF1. The patient’s father developed an oropharyngeal carcinoma, her paternal grandmother had a uterine carcinoma, and her paternal uncle was diagnosed with lung cancer (see **Figure 1A**). The patient therefore tentatively fulfilled two of the NIH diagnostic criteria (NIH, 1988)—axillary freckling and (possibly) two or more cutaneous neurofibromas. Overall, the patient’s clinical manifestations were somewhat atypical but compatible with the clinical diagnosis of NF1. Genetic testing was seen as potentially helpful in establishing the diagnosis unequivocally (Friedman, 1993).

After receiving the results of genetic testing, a revision of previous biopsies was performed at our institute—subcutaneous formations were confirmed to be lipomas, but the four fibroepithelial polyps were reclassified into neurofibromas based on their morphology and immunophenotype (a mixture of S100-positive spindle cells and CD34-positive stromal cells) (see **Supplementary Material**).

### Patient 2 (Harboring *NF1*:c.7395-17T > G)

The second patient was first seen at our cancer genetics clinic at the age of 42 with clinical diagnosis of NF1. She had previously been treated at our institute due to soft-tissue formations. One such tumor from her perianal region was diagnosed as a plexiform neurofibroma on histopathological examination. A tumor excised from the region of her left ankle was found to be an atypical neurofibroma. No additional treatment was necessary. She had also been diagnosed with a pelvic formation, most likely a plexiform neurofibroma, and was undergoing regular imaging to ascertain for possible growth.

On examination, she was found to have quite a typical clinical presentation for NF1, with pronounced disability caused by her disease. She had soft-tissue formations on her tongue, in the region of her left metacarpal bones, and on her left foot. Some of these tumors were growing and causing pain and weakness. She had multiple café-au-lait spots on her skin and axillary freckling, but had never undergone an ophthalmological examination; therefore, the presence of Lisch nodules could not be confirmed. She was diagnosed with cognitive impairment and received support from a special needs assistant but was able to live on her own. No macrocephaly was observed in our patient; her height was below average. In the patient’s family, her mother also had the clinical diagnosis of NF1 and died from a malignant peripheral nerve sheath tumor at the age of 47. No other relatives were reported to have had signs of NF1. The patient’s maternal aunt died of ovarian cancer aged 54 and her son (the patient’s cousin) was also reported to have died of malignant disease (see **Figure 1B**).

The patient’s clinical diagnosis of NF1 was clearly established as she fulfilled four of the NIH NF1 diagnostic criteria (6 or more café-au-lait spots, axillary freckling, confirmed plexiform neurofibroma, first-degree relative with a clinical diagnosis of NF1). Genetic testing was undertaken to confirm her diagnosis. Furthermore, we wanted to explore possible genotype–phenotype correlations.

## Materials and Methods

All procedures followed in this study were in accordance with the ethical standards of the responsible committee on human experimentation (institutional and national) and the Helsinki Declaration of 1975, as revised in 2000. Individual written informed consent from the participant for the publication of this case report was obtained as well as the institutional informed consent form for treatment, which included consent to use the patient’s data, materials, and/or test results for research purposes.

### DNA Extraction and NGS Sequencing

DNA from formalin-fixed paraffin-embedded tissue was extracted using QIAamp DNA FFPE Tissue Kit. Whole blood samples were collected into EDTA tubes. Genomic DNA was extracted using InnuPREP Master Blood kit (Analytik Jena, Thuringia, Germany). All exon regions and exon/intron boundaries ±25 nucleotides of NF1 and 93 other genes were enriched using Nextera DNA Library Preparation Kit in combination with commercially available TruSight Cancer Panel (Illumina, San Diego, USA), according to manufacturer’s protocol. Next generation sequencing was performed on Illumina MiSeqDx Sequencing System (Illumina). Read alignment to hg19 reference genome and variant calling was performed using MiSeq Reporter software. Variant annotation was performed using Variant Studio software and Alamut Visual software (Interactive Biosoftware, Rouen, France).

### MLPA

Multiplex ligation-dependent probe amplification (MLPA) from patients DNA was performed using SALSA MLPA P082 NF1 mix 1 and mix 2 (MRC-Holland, Amsterdam, Netherlands).

### RNA Extraction and Sanger Sequencing of cDNA

Blood from two patients and six healthy controls was collected into TempusBlood RNA tube and whole RNA was extracted using Tempus Blood Isolation kit (Thermo Fisher) following manufactures instructions. cDNA was reverse transcribed using ReverseScript RNA kit. To assess the pathogenicity of variants, RT-PCR followed by direct Sanger sequencing was performed. To asses pathogenicity of variant *NF1*:c.122A > T, primers aligning to 5’UTR and exon–exon junction of exons 4 and 5 were used. For variant c.7395-17T > G, primers flanking exons 48/49 and 53/54 were used. Primer sequences are available upon request.

### Transcript Quantification

Relative levels of mRNA transcripts from carriers and seven controls were assessed by capillary electrophoresis of FAM-labeled amplicons in a Genetic Analyzer ABI3500. Peak calling was performed with GeneMapper Software (Applied Biosystems). The splicing fraction for each alternative transcript was estimated as the ratio between the peak area of the individual transcript and the Σ of all peak areas (all transcripts) detected. Primers spanning 5’UTR and exon–exon junction of exons 4 and 5 were used for variant c.122A > T. For variant c.7395-17T > G, primers flanking exons 48/49 and 53/54 were used.

### Bioinformatic Prediction of Splicing Alteration

The possible effect on splicing was evaluated using Alamut Visual software 2.11 (Interactive Biosoftware, Rouen, France), which simultaneously executes six *in silico* splicing prediction tools: NNSplice (NNs), MaxEntScan (MET), Gene splicer, and SpliceSiteFinder-like (SSF).ESE prediction was performed using ESE-finder. The strength of an authentic splice site and *de novo* splice site was calculated as a mean of predicted splice site scores obtained from *in silico* tools: SSF, MET, and NNs.

The variants are described according to HGVS v19.01 nomenclature, using LRG_214t1 cDNA transcript (NM_000267.3). Both variants were submitted to ClinVar in October 2018 (SCV000839530 and SCV000840452), classified as likely pathogenic.

## Results

### *NF1* Genotyping

Two patients with clinically diagnosed neurofibromatosis type I were referred to our laboratory in order to perform genotyping of *NF1* gene. Genetic screening by targeted NGS was performed on the patients’ blood samples. After alignment of NGS reads and variant calling, SNVs that are common in the Slovenian population were filtered out. The remaining variants were discovered: a missense variant *NF1*:c.122A > T p.(Glu41Val) in patient 1 in exon 2 and an intronic variant in patient 2 *NF1*:c.7395-17T > G p.? in intron 50. Both variants are novel, previously not reported in the public databases or described in scientific literature (ClinVar, HGMD, and LOVD). Furthermore, both variants are rare, as they were not encountered in healthy populations (GnomAD and ExAc) nor in our cohort of more than 2,000 individuals who underwent germline testing.

MLPA screening of *NF1* gene was performed in order to exclude single or multiexon deletions or duplications and microdeletions. Both patients were negative for CNVs.

To better characterize the newly discovered variants, *in silico* prediction of their potential effect on mRNA splicing motifs was evaluated with four splicing prediction tools. The comparison was made between predicted wild type and mutated allele, summarized in **Table 1**. Three out of four splicing prediction tools (SSF, MET, and NNs) predicted the creation of a *de novo* splice site; 5’ ss by c.122A > T variant and a new 3’ ss by c.7395-17T > G.

**Table 1:** Bioinformatic evaluation of pathogenicity of two mutations by *in silico* splicing prediction analysis. For the analysis, SpliceSiteFinder-like, MaxEntScan, NNSplice, and Gene splicer were used. Mut-predicted strength of mutated allele ℕ-predicted strength of natural splice site.

DNA Variant	SpliceSite Finder-like		MaxEntScan		NNSplice		Gene splicer		% variation
	mut	ℕ	mut	ℕ	Mut	ℕ	mut	ℕ	
c.122A > T	89.52	86.82	9.79	10.13	1.00	1	–	–	+2.4
c.7395-17T > G	83.94	97.1	5.62	10.3	0.72	1	–	–	−20%

### Functional mRNA Analysis

**Figure 2:**
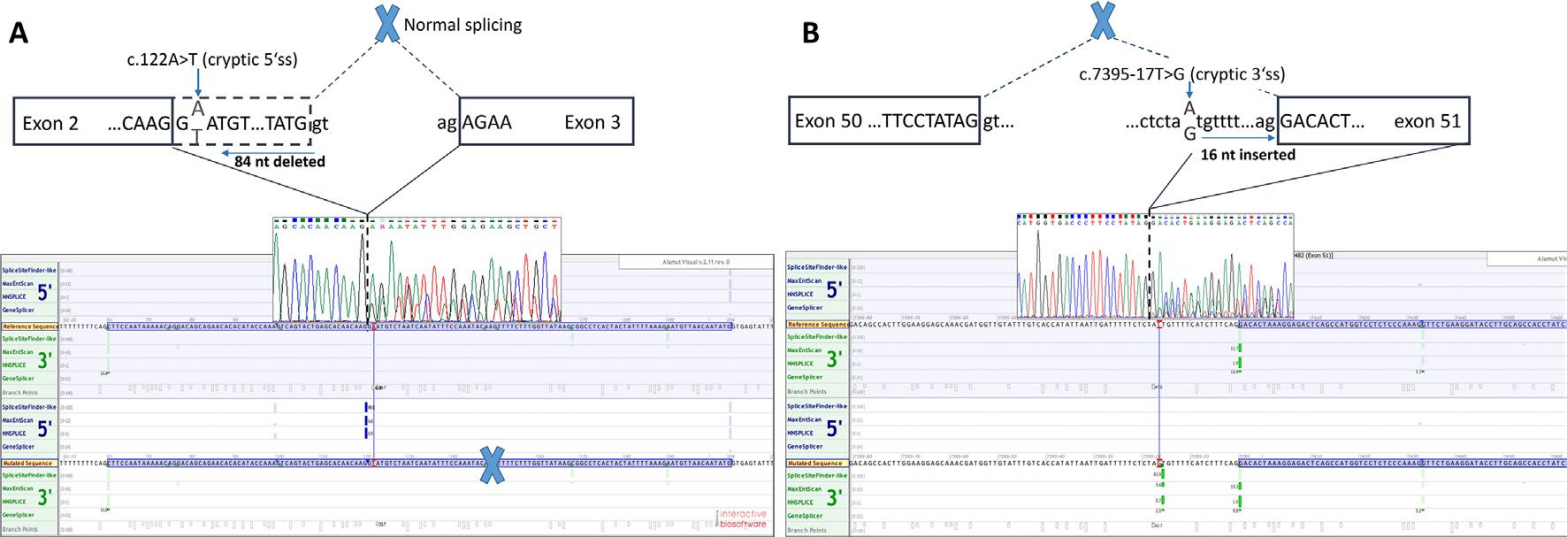
Two novel *NF1* mutations and their effect on mRNA splicing. **(A)** Sanger sequencing electropherogram and *in silico* splicing prediction of variants using Alamut Visual software for variant c.122A > T and **(B)** for variant c.7395-17A > G.

**Figure 3:**
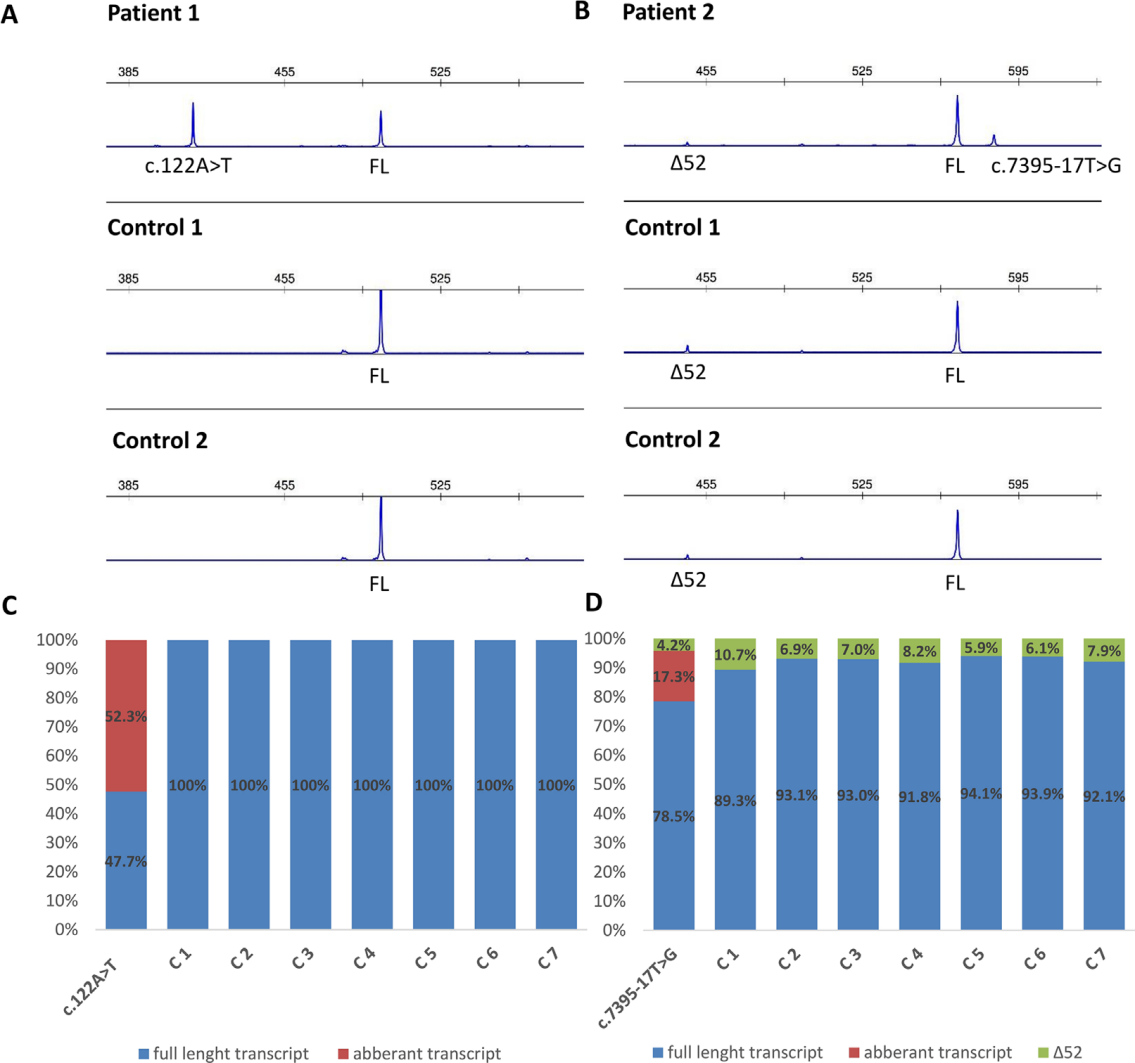
Fragment analysis profiles of **(A)** patient 1, carrier of *NF1*: c.122A > T and two controls (廼primers spanning 5’UTR and exon–exon junction of exons 4 and 5); **(B)** patient 2, carrier of *NF1*:c.7395-17T > G and two controls (primers flanking exons 48/49 and 53/54), showing the amount of abberant transcripts,alternative isoform lacking exon 52 (Δ52) and full-length transcript (FL). **(C, D)** Ratio between abberant and wild-type transcript for patients 1 and 2 measured by capillary electrophoresis analysis of FAM-labeled fragments.

Functional mRNA analysis was performed from patients’ RNA samples for evaluation of the pathogenicity of the variants c.122A > T and c.7395-17T > G with respect to mRNA splicing. Variant c.122A > T produced a new canonical splice site AG/GT, which lead to the deletion of last 84 bp of exon 2. Variant c.7395-17T > G introduced *de novo* 3’ss, which out-competed authentic 3’ ss, leading to retention of 16 intronic nucleotides; see schematical representation of both variants in **Table 2** and **Figure 2**. In both instances, the authentic polypyrimidine tract and branch site can be used. Six RNA samples obtained from individuals not carrying the variants in question, served as controls. Additionally, semiquantitative estimation of splicing isoforms obtained from capillary electrophoresis of FAM-labeled amplicons indicated that impaired transcript caused by c.7395-17T > G variant accounted for (17.3%) in comparison to full-length transcript (78.5%) and alternative transcript lacking exon 52 (4.17%). Altered transcript that is the product of c.122A > T variant, lacking 84 bp of exon 2, accounted for 52% in comparison to 48% of full-length transcript. All control samples lacked the mutated transcripts (**Figure 3**).

**Table 2:** Detected changes by Sanger sequencing on mRNA level and predicted change on protein level.

DNA variant	hg19 genomic coordinate	Effect on mRNA		Predicted protein
c.122A > T	Chr17:g.29483062A > T	New 5’ ss, deletion of last 84 nt	r.121_204del	p.(Glu41_Met68del)
c.7395-17T > G	Chr17:g.29679258T > G	New 3’ ss, insertion of 16 intronic nt	r.7394_7395ins7395-16_7395-1	p.(Arg2465Serfs*21)

### Segregational Studies

Segregational studies were hindered by a limited number of relatives available for testing. For patient 1, her healthy sister with no signs of neurofibromatosis was tested and shown not to be a carrier of the c.122A > T variant. The mother of patient 1 could not be tested for the presence of the *NF1* variant due to old age and poor general health. For patient 2, we performed a postmortem analysis using nontumor formalin-fixed paraffin-embedded tissue obtained from her affected mother. We were able to confirm the presence of the c.7395-17T > G variant in the mother’s sample.

## Discussion

Defining the pathogenicity of *NF1* variants enables appropriate genetic counseling and clinical management of neurofibromatosis type I patients and their relatives. However, testing for the presence of a *NF1* causative variant is challenging due to occurrence of unusual splice mutations outside of the conserved splice sites (Ars et al., 2000; Pros et al., 2008; Evans et al., 2016). In this study, we report the bioinformatical and functional evaluation of two novel *NF1* variants: c.122A > T and c.7395-17T > G. Both variants were found in neurofibromatosis type I patients. *In silico* analysis predicted disruption of normal splicing. Previous studies have reported that the predicted strengths of the new 3’ splice sites, generated due to mutations, are lower than those of the corresponding authentic splice sites (Wimmer et al., 2007; Jang et al., 2016). Concurring with published data, the predicted strength of the new acceptor splice site created by *NF1*:c.7395-17T > G variant was 21% lower than the strength of the corresponding authentic splice site. Interestingly, the estimated strength of authentic 5’ splice site (c.7395) was not drastically reduced. The predicted strength of the newly created exonic splice site created by *NF1*:c.122A > T variant was slightly (3%) greater than the strength of the authentic 5’ splice site (c.204).

The recognition of authentic splice site is not only based on intronic AG/GT splicing signals and branch point but also on splicing exonic enhancers (ESE) and silencers (ESS) (Cartegni et al., 2002). ESE and ESS play an essential role in determining alternative splicing. DNA variants that interrupt ESE motifs can result in exon skipping. On the contrary, variants can also introduce new ESE motif causing SR proteins to recognize the ESE motif stimulating the spliceosome machinery to recognize alternative exon, resulting in incorrect splice site recognition (Cartegni et al., 2002; Zatkova et al., 2004). A further *in silico* prediction of cis-acting elements, using ESE-finder, has revealed that the ESE sequence motif for our variants was altered. Gain of putative ESE was predicted for both variants: c.122A > T caused the gain of SRp55 motif; c.7395-17T > G created a new SRp40 motif. Both proteins SRp55 (SRSF6) and SRp40 (SRSF5) promote exon definition when bound to a corresponding ESE (Cartegni et al., 2002; Jeong, 2017; Juan-Mateu et al., 2018). We hypothesize that both variants not only introduce a new splice site but also enhance splicing by creation of an ESE and consequential binding of SR proteins. However, this hypothesis is based on *in silico* prediction tools and should therefore be confirmed with a functional assay. Indeed, the data obtained from patients mRNA confirmed that tested variants cause splicing impairment. Variants affecting splicing have been previously divided in five classes by Wimmer et al. (2007): type I, classical splicing variants affecting canonical +/−1,2 splice site inducing exon skipping; type II, intronic variants, causing the inclusion of cryptic exon into mRNA; type III variants that introduce *de novo* splice site in the exon, which leads to skipping of a part of an exon; type IV variants that strengthen cryptic splice site and disrupt canonical splice site; and type V variants occurring in exon and leading to exon skipping. Adopting classification of *NF1* splicing variants from Wimmer et al. (2007), we have categorized c.122A > T as type III variant and *NF1*:c.7395-17T > G as type IV variant. mRNA analysis has revealed that c.122A > T variant causes the deletion of 84 bp (r.121_204del), enabling the maintenance of reading frame. Transcript quantification disclosed that aberrant isoform lacking 84 bp accounted for 52% in comparison to 48% of full-length transcript, which is in concordance with the heterozygous state of the variant. Predicted effect on protein level is a deletion of 28 amino acids (AA), from Glu 41 to Met 68, which may cause misfolded and inactive protein. On the other hand, the deletion of 84 bp (28AA from 41 to 68) can result in altered mRNA secondary structure, which may interfere with ribosome binding and protein translation. In addition, no known protein binding sites are located from amino acid 41 to 68. However, some proteins (e.g., Kinesin-1, Cullin 3) are known to interact with neurofibromin, but their binding sites are unknown (Ratner and Miller, 2015). Therefore, the deletion of 28 amino acids could influence the neurofibromin interactions with other proteins. Moreover, this protein region shows interspecies conservation, since 17 of 28 (60.7%) amino acids are highly conserved up to fruit fly, suggesting the region’s importance. However, this is a speculation that should be verified by experimental data. Second, variant *NF1*:c.7395-17T > G resulted in the retention of last 16 bp of intron 50 (r.7394_7395ins7395-16_7395-1), leading to frameshift and introduction of premature stop codon at the amino acid residue 2465. However, aberrantly spliced mRNAs are subject to instability and nonsense medicated decay, and it is possible that mRNA is degraded and no protein is being produced. This hypothesis is in concordance with the fact that the isoform with intron retention accounts for only 17.3% in comparison to 78.5% of full-length isoform, even though it is present as a heterozygous variant. Based on the results of mRNA analysis, both variants were classified as likely pathogenic applying evidence categories PS3 (well established *in vitro* functional study support damaging effect), PM2 (both variants are absent in GnomAD controls), PP3 (computational evidence supports deleterious effect), PP1 supporting (both variants cosegregate with disease in affected family members) according to ACMG guidelines (Richards et al., 2015).

As a secondary finding, our mRNA analysis has revealed an alternative *NF1* mRNA isoform that lacks exon 52. Both probands and healthy controls were subject to an in-frame skipping of exon 52 (c.7553-c.7675; chr17:29683478-29683600). The alternative isoform was present in 7.5% in control samples and in 4.2% in carrier of *NF1*:c.7395-17T > G variant. Since this isoform was not only present in affected individuals but also in unaffected individuals, this isoform is normally present in blood, which was also previously published by Vandenbroucke et al. (2002) (describing this transcript as ∆43).

A multistep pathogenic variant detection protocol that combines analysis of genomic DNA and cDNA (mRNA) with testing for copy number changes is usually recommended for *NF1* molecular genetic testing (Evans et al., 2016). Most molecular genetic laboratories have gradually replaced Sanger sequencing with NGS-based testing methods (Stella et al., 2018), and some do not routinely perform cDNA analyses, since this approach is time consuming and labor intensive. Furthermore, there is a clinical overlap between certain RASopathies and NF1, and multigene testing may be warranted in such cases. We therefore propose an alternative approach whereby analysis of genomic DNA using NGS is performed first and targeted cDNA testing is done subsequently, to confirm the pathogenicity of potential splicing variants. In case no potentially causative variants are found with NGS in patents fulfilling the clinical criteria, additional cDNA-based analyses can be performed for detection of splicing variants outside targeted regions. Such an approach increases the sensitivity of testing in laboratories not specialized in *NF1* without greatly increasing the workload.

Patient 1 illustrates the fact that some cases of *NF1* can be difficult to diagnose even in adulthood. At first, her presentation was seen as atypical, with few café-au-lait spots and skin formations which were erroneously classified as fibroepithelial polyps. As the pathogenicity of her *NF1* variant was demonstrated and the results of previous biopsies revised, the diagnosis could finally be confirmed. It was also supported by the fact that she developed a spontaneous hemothorax, which is an extremely rare but possibly fatal complication of *NF1*. Since 1975, over 50 *NF1* cases with spontaneous hemothorax have been described in the literature. It appears to be more common in female and develops either due to vascular malformations leading to aneurisms or due to degeneration of thoracic neurofibromas (Gil Vázquez et al., 2018; Degbelo et al., 2019). The patient’s breast cancer may also be linked to her diagnosis of NF1 as the association between premenopausal breast cancer and NF1 is now widely recognized (Howell et al., 2017).

The phenotype in patient 2 was relatively severe. In addition, her mother had died of NF1-related complications. At the moment, data on genotype–phenotype correlations in NF1 are limited due to large numbers and diversity of *NF1* pathogenic variants and the importance of random factors in shaping the phenotype of affected individuals (Shofty et al., 2015; Xu et al., 2018). There is, however, some evidence that truncating variants are associated with solid malignancies and a more severe phenotype (Ponti et al., 2014).

In conclusion, the mRNA analysis is highly important in order to identify variants that have an effect on splicing. Using this approach, the two novel variants in *NF1* gene were successfully classified according to ACMG classification as likely pathogenic variants, confirming the neurofibromatosis type I phenotype in both probands. If *in silico* splicing prediction and mRNA analysis had not been performed, both variants described in this report could have been misclassified as harmless intronic and missense alterations. However, many challenges arise when performing mRNA analysis from patients’ blood samples. Detection of altered splice variants relays greatly on experiment design, since one variant does not only result in one mRNA species; numerous alternative mRNA transcripts are expressed in different tissues, which are frequently not described in public databases or presented as transcript reference sequences. Therefore, mRNA analysis is a complex, labor-intensive assay, but is crucial for variant interpretation.

## Data Availability

The datasets generated for this study can be found in ClinVar, SCV000840452, SCV000839530.

## Ethics Statement

This study was carried out in accordance with the recommendations of Slovenian National Medical Ethics Committee with written informed consent from all subjects. All subjects gave written informed consent in accordance with the Declaration of Helsinki. The protocol was approved by the Slovenian National Medical Ethics Committee.

## Author Contributions

VSD wrote the manuscript and performed the experiments. MK collected clinical data. AB wrote the clinical part of the manuscript. GK and VS performed NGS analysis. OB performed pathological revision. SN wrote, reviewed, and edited the manuscript.

## Funding

The study was funded from Slovenian research agency, program number: P3-0352.

## Conflict of Interest Statement

The authors declare that the research was conducted in the absence of any commercial or financial relationships that could be construed as a potential conflict of interest.

## Supplementary Material

Supplementary Figure 1 Patient’s 1 H&E staining (20×) of the lesion primarily diagnosed as fibroepithelial polyp and after revision reclassified as neurofibroma. The lesion is composed of a mixture of scarce spindle cells with wavy nuclei and fibroblasts. In the upper left corner, a basal portion of the epidermis is seen.
