# Supplementary figures and images for: Two Novel NF1 Pathogenic Variants Causing the Creation of a New Splice Site in Patients With Neurofibromatosis Type I
Source: Front Genet. 2019 Aug 22;10:762. doi: 10.3389/fgene.2019.00762 (PMC6714493; doi:10.3389/fgene.2019.00762)

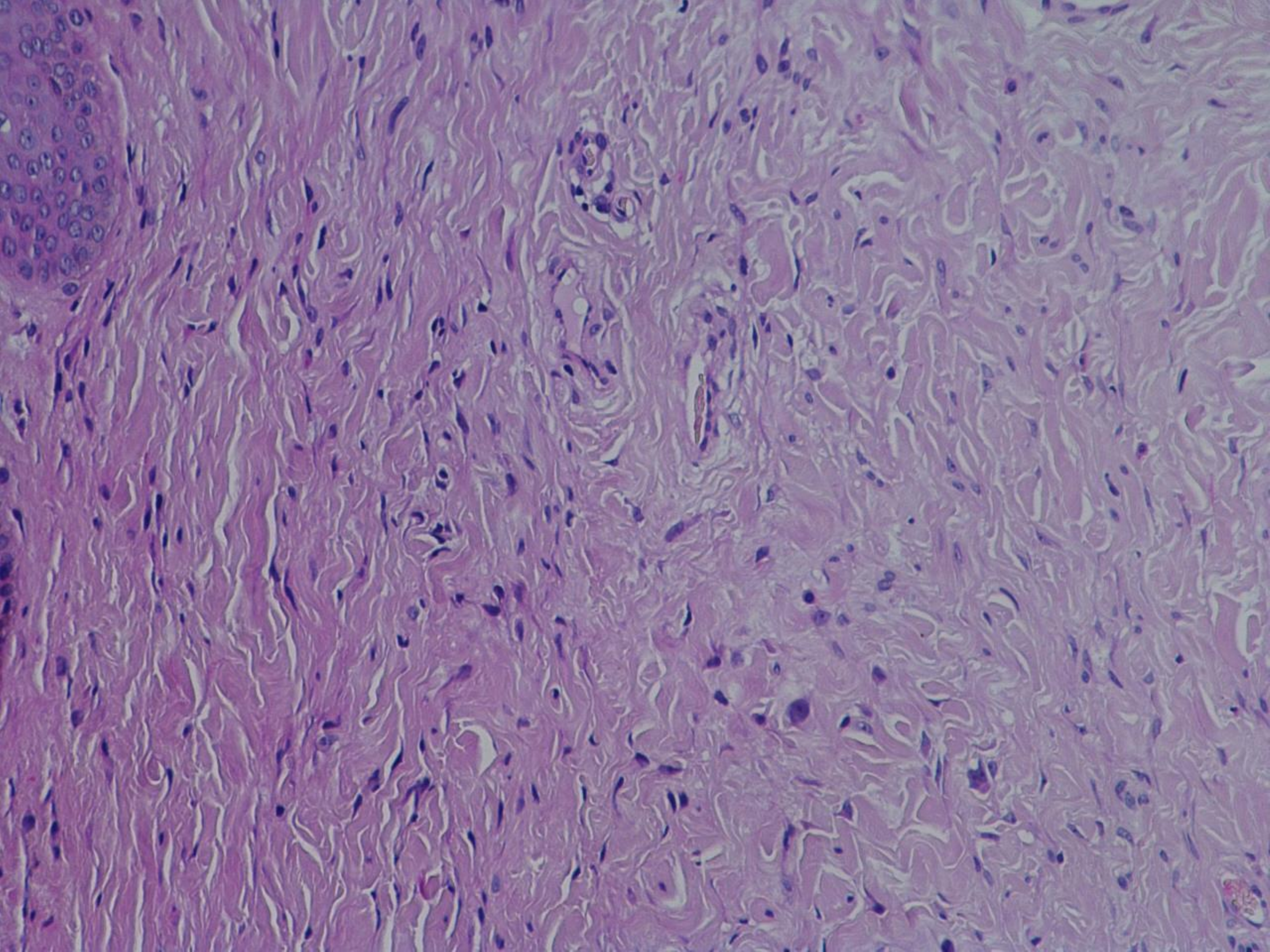

Supplement: Supplementary Figure 1 — Patient’s 1 H&E staining (20×) of the lesion primarily diagnosed as fibroepithelial polyp and after revision reclassified as neurofibroma. The lesion is composed of a mixture of scarce spindle cells with wavy nuclei and fibroblasts. In the upper left corner, a basal portion of the epidermis is seen. [file Image_1.jpeg]
